# NLRP1 Inflammasome Activation in the Hippocampal Formation in Alzheimer’s Disease: Correlation with Neuropathological Changes and Unbiasedly Estimated Neuronal Loss

**DOI:** 10.3390/cells11142223

**Published:** 2022-07-17

**Authors:** Ena Španić, Lea Langer Horvat, Katarina Ilić, Patrick R. Hof, Goran Šimić

**Affiliations:** 1 Department of Neuroscience, Croatian Institute for Brain Research, University of Zagreb School of Medicine, Šalata 12, 10000 Zagreb, Croatia; ena.spanic@hiim.hr (E.Š.); lea.langer@yahoo.co.uk (L.L.H.); katarinna.ilic@gmail.com (K.I.); 2 BRAIN Centre, Institute of Psychiatry, Psychology, and Neuroscience, King’s College London, London WC2R 2LS, UK; 3 Nash Family Department of Neuroscience, Friedman Brain Institute, and Ronald M. Loeb Center for Alzheimer’s Disease, Icahn School of Medicine at Mount Sinai, New York, NY 10029, USA; patrick.hof@mssm.edu

**Keywords:** Alzheimer’s disease, hippocampus, inflammasome, microglia, neurofibrillary pathology, NLRP3, tau protein

## Abstract

Neuroinflammation is one of the core pathological features of Alzheimer’s disease (AD) as both amyloid β (Aβ) and tau monomers and oligomers can trigger the long-term pro-inflammatory phenotype of microglial cells with consequent overactivation of the inflammasomes. To investigate the NLRP1 inflammasome activation in AD, we analyzed the expression of NLRP1, ASC, cleaved gasdermin (cGSDMD), and active caspase-6 (CASP-6) proteins in each hippocampal subdivision (hilar part of CA3, CA2/3, CA1, subiculum) of postmortem tissue of 9 cognitively healthy controls (HC) and 11 AD patients whose disease duration varied from 3 to 7 years after the clinical diagnosis. The total number of neurons, along with the total number of neurofibrillary tangles (NFTs), were estimated in Nissl- and adjacent modified Bielschowsky-stained sections, respectively, using the optical disector method. The same 9 HC and 11 AD cases were additionally semiquantitatively analyzed for expression of IBA1, HLA-DR, and CD68 microglial markers. Our results show that the expression of NLRP1, ASC, and CASP-6 is present in a significantly greater number of hippocampal formation neurons in AD brains compared to controls, suggesting that the NLRP1 inflammasome is more active in the AD brain. None of the investigated inflammasome and microglial markers were found to correlate with the age of the subjects or the duration of AD. However, besides positive correlations with microglial IBA1 expression in the subiculum and with microglial CD68 expression in the CA1 field and subiculum in the AD group, the overall NLRP1 expression in the hippocampal formation was positively correlated with the number of NFTs, thus providing a causal link between neuroinflammation and neurofibrillary degeneration. The accumulation of AT8-immunoreactive phosphorylated tau proteins that we observed at nuclear pores of large pyramidal neurons of the Ammon’s horn further supports their role in the extent of neuronal dysfunction and degeneration in AD. This is important because unlike fibrillar amyloid-β deposits that are not related to dementia severity, total NFTs and neuron numbers in the hippocampal formation, especially in the CA1 field, are the best correlates of cognitive deterioration in both human brain aging and AD. Our findings also support the notion that the CA2 field vulnerability is strongly linked to specific susceptibilities to different tauopathies, including primary age-related tauopathy. Altogether, these findings contrast with reports of nonsignificant microglial activation in aged nonhuman primates and indicate that susceptibility to inflammasome activation may render the human brain comparatively more vulnerable to neurodegenerative changes and AD. In conclusion, our results confirm a key role of NLRP1 inflammasome in AD pathogenesis and suggest NLRP1 as a potential diagnostic marker and therapeutic target to slow or prevent AD progression.

## 1. Introduction

Microglia are the most abundant immunocompetent cells in the brain. These brain-residing macrophages mediate nonspecific, innate immunity, and play important roles in the maintenance of homeostasis and neuroinflammation [1,2]. From the earliest descriptions of neuropathological changes in Alzheimer’s disease (AD), neuroinflammation has been one of the noted core features (for review, see [3]). Numerous studies have indicated that pathogenic forms of both amyloid β (Aβ) and tau protein monomers and oligomers can trigger the activation of microglial cells and induce their long-term proinflammatory phenotype [4,5,6,7,8,9,10,11,12]. However, in contrast to older paradigms where microglial activation had been considered as a mere biological response to Aβ accumulation, recent findings indicate that inflammation may parallel or even precede the development of AD pathological changes [13,14,15,16]. It is not known what initiates such changes, but it is presumed that disruption of the homeostatic levels of inflammatory mediators and neuroprotective molecules leads to chronic, uncontrolled neuroinflammation [17]. One of the possible mechanisms of chronic inflammatory response in the prodromal stages of AD could be the overactivation of the inflammasomes.

Inflammasomes are associated with inflammatory caspases 1, 4, 5, 11, and 12, and mediate some of the main programmed cell-death pathways [18]. Inflammasomes are supramolecular protein complexes that assemble in the cytosol in response to pathogens, noxious substances, metabolic perturbations (e.g., increased levels of free fatty acids and mitochondrial reactive oxygen species), and after sensing various damage-associated signals within cytoplasm through nucleotide-binding and oligomerization domain (NOD) of the NOD-like leucine-rich repeat receptors (NLRs) [19]. NLR family pyrin domain-containing 1 (NLRP1) is a 1429 amino-acid-long protein, known to form an inflammasome complex and activate caspase-1 upon degradation of its N-terminal part by the proteasome in neurons, whereas NLRP3 (NLR family pyrin domain-containing 3 protein) is the main NLRP family member in the brain, predominantly expressed in microglia. NLRP3 is a tripartite protein, consisting of an N-terminal pyrin domain (PYD), a central NOD, and a C-terminal leucine-rich repeat motif [20]. To activate caspase-1, both NLRP1 and NLRP3 inflammasomes recruit the adaptor protein ASC (apoptosis-associated speck-like protein containing a Caspase-Activation and Recruitment Domain), encoded by the *PYCARD* gene [18]. It is believed that ASC has a pivotal role in inflammasome assembly and activation. Once assembled, this inflammasome complex functions as an upstream activator of the NF-κB signaling pathway, which is known to play a key role in the regulation of inflammation, the innate immune response, and apoptosis [21]. Active caspase-1 (CASP-1) promotes proteolytic maturation of IL-1β and IL-18 and cleaves the gasdermin D (GSDMD) with the consequent formation of plasma membrane pores that lead to pathological ion fluxes resulting in pyroptotic cell death and release of inflammatory cytokines [18]. CASP-1 is also responsible for the CASP-6 activation [22] as cleavage of the CASP-6 in neurons is occurring downstream of the NLRP1 inflammasome activation [23]. Active CASP-6 is associated with AD pathological changes [24] and is present in all pathological alterations of AD, neurofibrillary tangles (NFTs), neuropil threads, and neuritic plaques, as well as pretangles [25,26]. It is also detectable inside neurons with or without NFTs [26]. In addition, intraneuronal CASP-6 activation increases with the progression of the disease [26].

The best-studied inflammasomes in AD are NLRP3 and NLRP1 [27,28,29,30,31,32,33,34]. Many AD patients have altered, proinflammatory gut microbiota, which is not detrimental just to the intestinal barrier, but can disrupt the blood-brain barrier, contributing to the reactive microglial response in the brain [35]. Gut dysbiosis induces NLRP3 inflammasome activation [35,36], and transplantation of a healthy gut microbiota into a rat model with AD-like pathology was shown to attenuate neuroinflammation and reduce Aβ, tau, and NLRP3 expression [37]. Inflammasomes are also highly expressed in other cells of the central nervous system. Unlike NLRP3, the NLRP1 inflammasome is predominantly expressed in pyramidal neurons and oligodendrocytes [38]. Intriguingly, in an Italian cohort of 542 subjects, four out of nine selected nonsynonymous *NLRP1* polymorphisms were found to be associated with AD [28]. Moreover, the expression of the NLRP1 increases upon the addition of Aβ in neuronal cell cultures and is increasingly expressed in several animal models of AD [30]. Likewise, silencing the *NLRP1* gene improves cognitive abilities and has a protective effect on neurons in animal models of AD [30,39,40,41,42].

Compared to controls, hippocampal NLRP1 immunoreactivity is higher in AD brains, whereas the NLRP1 inflammasome stress-induced activation in vitro results in elevated Aβ levels as a part of an acute protective response [23]. Therefore, cellular stress caused by various agents in sporadic AD or mutations of the genes in familial AD cases may trigger NLRP1 inflammasome activation, and further, promote an inflammatory response through astrocytes and microglia interplay [23].

Although these findings suggest that excessive NLRP1 inflammasome activation could contribute to the development and progression of AD, probably through its prodromal stage, the actual causes of its overactivation and exact chronology of pathological changes are not known. The present study provides a comparison of the NLRP1 inflammasome activation in the hippocampal formation (HF) of autopsy samples from AD and healthy controls (HC).

## 2. Materials and Methods

### 2.1. Human Brain Tissue Autopsy Samples

Archival human postmortem brain samples from 9 AD and 11 cognitively HC cases were selected from the Huddinge Brain Bank (Huddinge University Hospital, Stockholm). This tissue was chosen because of the extensive neuropathological characterization. The demographic data on the AD and control groups are shown in Table 1. The clinical diagnosis of AD was based on combined DSM-IV and NINCDS-ADRDA criteria [43], while the final neuropathological diagnosis was based on CERAD criteria [44]. All subjects in the control group died of non-neurological causes, and thioflavin-S, Congo red, Bielschowsky silver staining, and anti-tau immunohistochemistry showed no AD lesions nor lesions consistent with age-related changes.

Brain tissue was fixed in 4% paraformaldehyde buffered with 0.1% phosphate buffer within 24 h after death. For counting the number of neurons and NFTs, hippocampal tissue was sampled in a systematic-random manner: the left hippocampus was removed from each brain and cut in the rostrocaudal direction into 3 mm-thick blocks, with a random position for the first cut within the first rostral interval. Each block was dehydrated through a graded series of ethanol solutions, paraffin-embedded, and sectioned at 12 μm for staining. For assessment of inflammasome markers, a single random section was taken from the random block taken from the hippocampal body.

### 2.2. Immunohistochemical and Immunofluorescent Staining

Tissue sections were deparaffinized in xylene and rehydrated in the decreasing concentrations of ethanol (100%–twice, 96%, and 70%). Antigen retrieval was performed in a boiling citrate buffer (anhydrous citric acid solution 10 mM, pH 6), 5 times short (around 1 min) at high microwave power (700 W) and 20 min at low microwave power (300 W). Endogenous peroxidase activity was inhibited by incubating slides in 0.02% H_2_O_2_ in methanol (150 mL methanol and 50 mL water) for 30 min. Unspecific signal was blocked with 5% bovine serum albumin (BSA) + 0.5% Triton/PBS for 1 h at RT. Primary antibodies (NLRP1, Abcam, AB_776633; ASC, Invitrogen, AB_2804676; GSDMD, Cell Signaling, AB_2799099; Caspase-6, Antibodies-online, AB_2290879; IBA1, FUJIFILM Wako Shibayagi, AB_839504; CD68, Agilent, AB_2314148; HLA-DR, Agilent, AB_2313661) were diluted in blocking solution to working concentrations (NLRP1 1:100, ASC 1:100, GSDMD 1:500, caspase-6 1:100, IBA1 1:250, CD68 1:1250 and HLA-DR 1:300). After overnight incubation with primary antibodies in a humidified chamber at 4 °C, slides were incubated in the goat antirabbit or antimouse (for CD68 and HLA-DR) biotinylated secondary antibody (1:200) for 60 min (Vector Laboratories, Newark, CA, USA, AB_2336810, AB_2336811) followed by the application of the ABC complex also for 60 min at RT (Vector Laboratories, AB_2336810, AB_2336811). 3,3′-diaminobenzidine (Sigma, cat. #D0426) was used as chromogen for developing the peroxidase activity. Negative-control sections were not incubated in the primary antibodies. Sections were dehydrated before mounting in Histomount (Poly-Mount, Catalog #08381-120). Double-labeling immunofluorescence experiments were also performed. Deparaffinization, rehydration, and antigen-retrieval steps were performed as described above. Nonspecific binding was blocked with 1% BSA in 0.5% Triton/PBS for 1 h at RT. Primary antibodies (NLRP1, Abcam, AB_776633; ASC, Invitrogen, AB_2804676; Caspase-6, Antibodies-online, AB_2290879; CD68, Agilent, AB_2314148; HLA-DR, Agilent, AB_2313661; AT8, Thermo Fisher, AB_223647) were diluted in blocking solution to working concentrations (NLRP1 1:100, ASC 1:100, caspase-6 1:100, CD68 1:1250, HLA-DR 1:300, and AT8 1:200). Sections were incubated at 4 °C overnight. After washing, slides were incubated in the appropriate secondary goat antirabbit or antimouse antibodies conjugated with fluorophores (AlexaFluor TM AF488 goat, anti-mouse IgG, Thermo Fisher Scientific, Waltham, MA, USA, AB_2534088; AlexaFluor AF488 goat, anti-rabbit IgG, Thermo Fisher Scientific, AB_2576217; AlexaFluor AF546 goat, anti-mouse IgG, Thermo Fisher Scientific, AB_2534089; AlexaFluor AF546 goat, anti-rabbit IgG, Thermo Fisher Scientific, AB_2534093) for 2 h at RT before application of TrueBlack lipofuscin Autofluorescence quencher (5 μL TrueBlack + 100 μL 70% EtOH) 45 s per sample. After washing, samples were covered with the mounting medium with 4′,6-diamidino-2-phenylindole (DAPI, Vectashield Antifade Mounting Medium with DAPI) and imaged on the confocal microscope Olympus FV3000 (Tokyo, Japan).

### 2.3. Analysis of Immunohistochemically Stained Sections

Quantification was performed by E.Š., who was blind to the experimental group and the identity of the cases. Tissue-section analysis and images of the slides were obtained with an Olympus BX53 microscope (Olympus, Tokyo, Japan). The total number of all immunoreactive cells in a section randomly selected prior to staining and taken from the hippocampal body and the four hippocampal subfields were analyzed (hilar region of the CA3, CA2/3, CA1, and subiculum) using Image J software (National Institutes of Health, Bethesda, MD, USA, https://imagej.nih.gov/ij/ [accessed on 16 December 2021]). The results were presented as the number of immunoreactive cells per region of interest. All three microglial markers were analyzed by E.Š., K.I., and L.L.H. Microglial markers IBA1 and HLA-DR were analyzed semiquantitatively according to the following scale: 0—immunoreactivity is not present; 1—several immunoreactive cells are present, all cells are ramified microglia; 2—moderate number of immunoreactive cells, mostly ramified, few activated cells; 3—many diffusely distributed immunoreactive cells, mostly activated; and 4—many large clusters of activated microglial cells. Microglial marker CD68 was analyzed semiquantitatively according to the following scale: 0—immunoreactivity is not present; 1—several immunoreactive cells are present; 2—moderate number of immunoreactive cells; 3—many diffusely distributed immunoreactive cells; and 4—many large clusters of immunoreactive microglial cells.

### 2.4. Unbiased Quantification of Neurons and Neurofibrillary Tangles

Quantification was performed by G.Š., who was blind to the experimental group and the identity of the cases. The total number of neurons and neurofibrillary tangles (NFTs) in each hippocampal subdivision was estimated in Nissl- and adjacent modified Bielschowsky-stained sections (Bielschowsky-staining modification according to Yamamoto and Hirano [45]), respectively, using the optical disector method, as described previously in detail [46]. In short, using an automated Olympus Video Stereological Analysis System (BICO, Copenhagen, Denmark) by using a low-power magnification we first delineated the subfields of the HF. Then, estimates of the reference volume of the delineated subdivisions were made using the Cavalieri principle, after correction for the shrinkage due to histological processing. For the determination of the shrinkage in the 3rd dimension, we used the value of the squared root of the previously determined areal (2-dimensional) shrinkage as a correction factor on slab thickness. The average value of shrinkage for Nissl-stained sections of HC cases was on average 18% (SD = 8.5), for AD cases 23.6% (SD = 7.2), and overall 20.8% (SD = 8.2). The average value of shrinkage for Bielschowsky-stained sections of HC cases was on average 18.1% (SD = 9), for AD cases 24.9% (SD = 8.2), and overall 21.5% (SD = 9.1). The second step was measuring the numerical density of neurons and NFTs by using the disector method [47]. An estimate of numerical density within an individual with a predetermined coefficient of error of less than 0.10 was achieved with about 100 observations per one field in one hippocampus performed in a systematic-random manner using a stepping meander path function. Assuming that all cells have one, and only one, nucleus, estimates of the number of neurons were based on counting nuclei in Nissl-stained sections at high magnification using an ×100 oil immersion objective with high numerical aperture, the appropriate superimposed counting frames of variable size (90 × 90 µm for subiculum and hilar part of the CA3 field, 60 × 60 µm for CA1 and CA2/3). The disector height was set at 10 µm. Neurons for which the clearest nuclear profiles fell within the disector volume and did not touch the left and bottom borders of the superimposed counting frames nor the superior “look-up” plane were counted, whereas NFTs were counted if characteristic silver-staining positive structures fulfilled the same criteria in modified Bielschowsky-stained sections [48]. Finally, the total numbers of neurons and NFTs were obtained by multiplying the numerical density of the particular hippocampal subdivision with its reference volume.

### 2.5. Statistical Analysis

Because we had a relatively small sample size in both AD and HC groups and the median value better represented the center of distribution for most of the investigated variables, we used the nonparametric Mann–Whitney U test and reported a two-tailed p-value, whereas correlations were performed using Spearman’s correlation coefficient r_S_ and correlation test. In those cases where analyzed variables were unbiasedly estimated using the optical disector method, such as the number of neurons and the number of NFTs, and the data were normally distributed, we used Student’s *t*-test, and Pearson’s correlation coefficient r_P_, and correlation test. The level of statistical significance in all tests was set at α = 0.05. All statistical tests and graphs were made in GraphPad Prism version 9.3.1. (GraphPad Software, San Diego, CA, USA).

## 3. Results

### 3.1. Analysis of NLRP1, ASC, cGSDMD, and Caspase-6 Immunostaining

**Figure 1:**
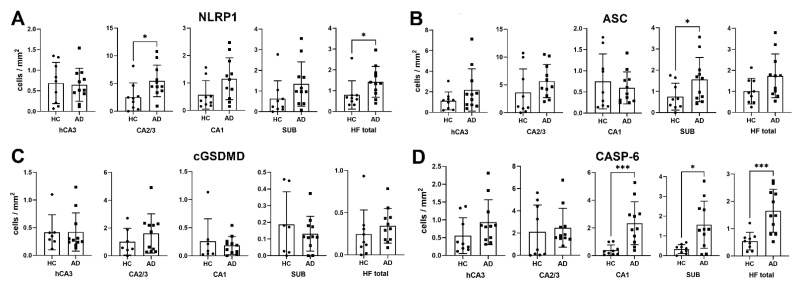
The regional density of immunoreactive neurons for NLRP1 (**A**), ASC (**B**), cGSDMD (**C**), and CASP-6 (**D**). (**A**) Compared to HC, a significantly higher quantity of NLRP1-labeled cells was found in the CA2/3 field in the AD group (*p* = 0.02). Although individual comparisons of hCA3, CA1, and SUB were not significant (*p* = 0.88, *p* = 0.11, and *p* = 0.07, respectively), when the hippocampal formation (HF) was viewed as a whole, the difference in the quantity of NLRP1-labeled cells was statistically significant (*p* = 0.03). (**B**) Compared to HC, a significantly higher quantity of ASC-labeled cells was found in the SUB in the AD group (*p* = 0.04). Other individual comparisons of ASC-labeled cells in the hCA3, CA2/3, and CA1 fields were not significant (*p* = 0.18, *p* = 0.13, and *p* = 0.99, respectively) as was the hippocampal formation (HF) viewed as a whole (*p* = 0.07). (**C**) Regarding cGSDMD, in the AD group neither the hCA3, CA2/3, CA1, and subiculum were significantly different from HC (*p* = 0.6, *p* = 0.54, *p* = 0.79, *p* = 0.72, respectively) nor was the hippocampal formation (HF) viewed as a whole (*p* = 0.15). (**D**) Regarding CASP-6 labeling, a significantly higher quantity of labeled cells was found in the CA1 and subiculum in the AD group compared to HC (*p* = 0.0005 and 0.02, respectively). Comparisons of hCA3 and CA2/3 were not significant (*p* = 0.13 and *p* = 0.41, respectively). When the hippocampal formation (HF) is viewed as a whole, the difference was highly significant (*p* = 0.0008). * = *p* < 0.05, *** = *p* < 0.001.

Results of the NLRP1, ASC, cGSDMD, and CASP-6 immunostaining assessment are shown in Table 2. Compared to the HC group, NLRP1 immunoreactivity was found in the significantly greater number of the CA2/3 neurons of the AD group (*p* = 0.02) and also when all fields in both groups were analyzed together (HF total, *p* = 0.03, Figure 1A). The ASC immunoreactivity was observed in the significantly greater number of neurons in the AD group in the subiculum only (*p* = 0.04, Figure 1B). The cGSDMD immunoreactivity was the weakest of all markers analyzed and also not significantly different in the number of immunoreactive neurons between the HC and the AD groups in any of the HF fields (Figure 1C). The CASP-6 immunoreactivity was observed in a significantly higher number of HF neurons (*p* = 0.0008), especially in the CA1 (0.0005) and subiculum (*p* = 0.02, Figure 1D).

### 3.2. Correlations between Immunohistochemical Markers of Inflammasome Activation

**Figure 2:**
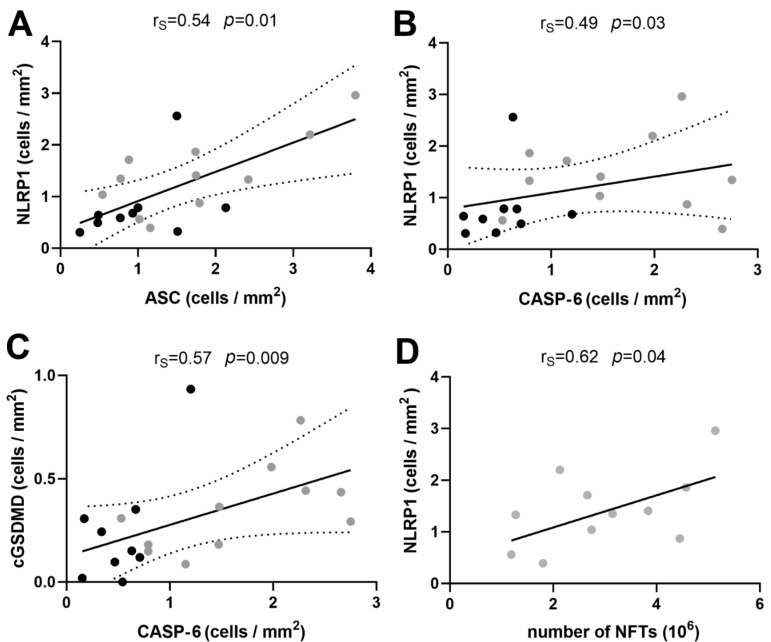
(**A**) Correlation between NLRP1 and ASC immunoreactivity. (**B**) Correlation between NLRP1 and CASP-6. (**C**) Correlation between cGSDMD and CASP-6. Dotted lines represent the 95% confidence interval. (**D**) The total number of NLRP1-immunoreactive neurons per area of hippocampal formation tissue significantly positively correlated with the total number of NFTs.

We found a significantly positive correlation between the overall numbers of HF neurons immunoreactive for NLRP1 and ASC per unit of tissue (Spearman’s coefficient of correlation r_S_ = 0.54, *p* = 0.01, Figure 2A) as well as between the numbers of HF neurons immunoreactive for NLRP1 and CASP-6 per unit of tissue (r_S_ = 0.49, *p* = 0.03, Figure 2B). While the correlation between ASC and CASP-6 was weak (r_S_ = 0.41) and not significant (*p* = 0.07) as well as the correlation between ASC and cGSDMD (r_S_ = 0.44, *p* = 0.053), the correlation between the total number of CASP-6 immunoreactive neurons per squared millimeter of HF tissue significantly positively correlated with the total number of cGSDMD immunoreactive neurons (r_s_ = 0.57, *p* = 0.009, Figure 2C).

### 3.3. Correlation of Immunohistochemical Markers with the Age of the Subjects and Duration of Disease

We correlated the immunostaining analyses of all four markers (Table 3) with the age of the subjects and the duration of AD. Neither of the markers correlated with the age of the subjects (NLRP1 r_S_ = 0.05, *p* = 0.84; ASC r_S_ = 0.14, *p* = 0.55; cGSDMD r_S_ = −0.06, *p* = 0.79; CASP-6 r_S_ = −0.03, *p* = 0.44). Likewise, no marker had a correlation with the duration of the disease (NLRP1 r_S_ = 0.52, *p* = 0.1; ASC r_S_ = 0.45, *p* = 0.17; cGSDMD r_S_ = 0.42, *p* = 0.19; CASP-6 r_S_ = 0.11, *p* = 0.76).

### 3.4. Correlations between NLRP1 Inflammasome, Neurofibrillary Tangles, Number of Neurons, and Disease Duration

The proportion of the number of NFTs and the number of neurons (Table 4) in the AD group was in the relatively narrow band of values (mean ± SD: hCA3 0.17 ± 0.14, CA2/3 0.22 ± 0.11, CA1 0.52 ± 0.22, subiculum 0.28 ± 0.19). Interestingly, this proportion did not correlate with the duration of the disease (hCA3 r_P_ = −0.38, *p* = 0.35; CA2/3 r_P_ = 0.2, *p* = 0.64; CA1 r_P_ = 0.35, *p* = 0.29; subiculum r_P_ = −0.36, *p* = 0.28), meaning that there might be some ‘fixed’ number of hippocampal neurons that are predisposed and will develop neurofibrillary pathology. The total number of NLRP1-immunoreactive neurons per area of hippocampal formation tissue significantly positively correlated with the total number of NFTs (Figure 2D).

### 3.5. Age-Related Neuronal Loss and Number of Neurofibrillary Tangles

**Figure 3:**
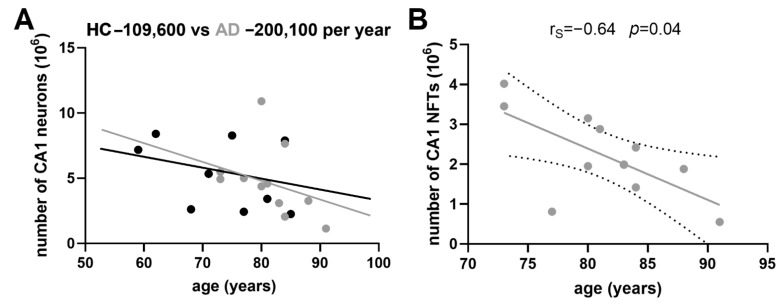
(**A**) Age-related neuronal loss in the CA1 field of HC and AD cases. The average annual difference is 90.5 thousand neurons. (**B**) The age-related number of NFTs negatively correlates with the age of AD subjects in the CA1 field of hippocampal formation. This means that younger AD patients had more neurofibrillary tangles than the older ones. Dotted lines represent the 95% confidence interval.

The slope of age-related neuronal loss was much steeper in AD patients than in HC for all the fields analyzed. The average negative difference was: −44.5 for the hCA3, −35.1 for the CA2/3 field, −90.5 for the CA1 field (−109.6 in HC vs. −200.1 in AD, Figure 3A), and −40.5 for the subiculum (all numbers are in thousands of neurons per year). The number of NFTs did not correlate with the age of AD subject in any of the HF fields analyzed (hCA3 r_S_ = 0.33, *p* = 0.32; CA2/3 r_S_ = 0.14, *p* = 0.67; subiculum r_S_ = −0.16, *p* = 0.65), except in the CA1 field, where it significantly negatively correlated with age (r_S_ = −0.64, *p* = 0.04), meaning that younger AD patients had more NFTs in the CA1 field than older ones (Figure 3B).

### 3.6. Assessment of Microglial Markers

#### 3.6.1. IBA1

**Figure 4:**
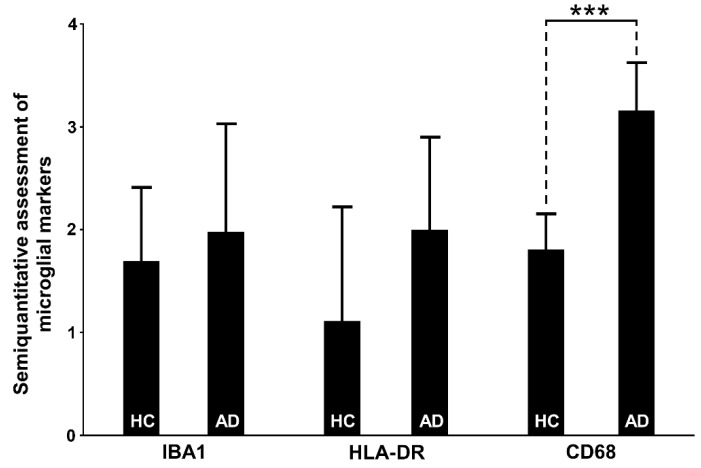
Semiquantitative assessment of IBA1/HLA-DR/CD68 immunoreactive microglial cells per randomly selected section in healthy control (HC) and Alzheimer’s disease (AD) subjects across the hippocampus. Microglial markers were analyzed for IBA1 and HLA-DR according to the following scale: 0—immunoreactivity is not present; 1—several immunoreactive cells are present, all cells are ramified microglia; 2—moderate number of immunoreactive cells, mostly ramified, few activated cells; 3—many diffusely distributed immunoreactive cells, mostly activated; and 4—many large clusters of activated microglial cells; marker CD68 was analyzed according to the following scale: 0—immunoreactivity is not present; 1—several immunoreactive cells are present; 2—moderate number of immunoreactive cells; 3—many diffusely distributed immunoreactive cells; and 4—many large clusters of immunoreactive microglial cells. CD68 expression is significantly higher in the AD group compared to HC (T = −7.22, d.f. = 18, with pooled estimate of variance *p* < 0.001). Data are represented as means ± SD. *** = *p* < 0.001.

**Figure 5:**
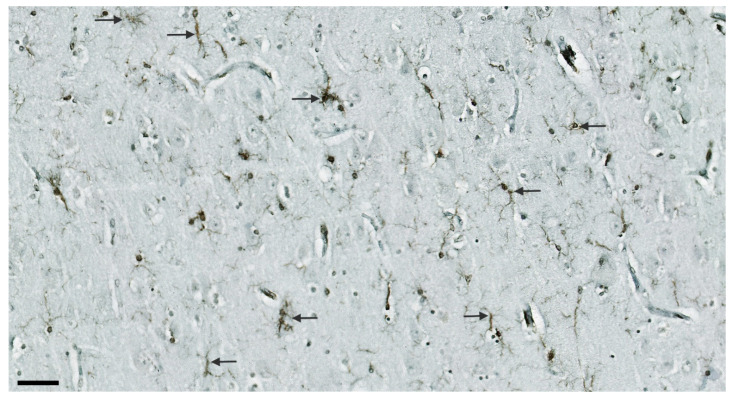
Expression of IBA1 marker in ramified microglia (arrows) in the CA1 in AD (case AD5). Scale bar 50 µm.

Despite the observed qualitative differences, the IBA1 marker did not show a significant difference in any of the HF fields between the AD and HC groups, nor when testing the total sum of all positive microglial cells per sample (*p* = 0.23; Table 5, Figure 4). However, we found that the degree of semiquantitatively-assessed expression of IBA1-expressing microglia correlated positively with the CD68-immunoreactive microglia (r_S_ = 0.55, *p* = 0.02). IBA1 expression positively correlated with the NLRP1 in the subiculum (r_s_ = 0.47, *p* = 0.04) and negatively with the ASC in hCA3 (r = −0.46, *p* = 0.04). In our samples, the IBA1 marker predominantly stained ramified microglia (Figure 5). The staining was not so intense and did not show large clusters as in the case of CD68 or HLA-DR. Only exceptionally, the irregularly ramified, ameboid microglial cells were stained with IBA1. In the HC group, IBA1 was most pronounced in the hCA3, whereas in the AD group the greatest number of IBA11-expressing microglial cells was also found in the CA2/3. The lowest numbers of IBA1-immunopositive microglia in the HC group were found in the CA1 and subiculum, whereas the lowest values for IBA1 in AD were found in the hCA3.

#### 3.6.2. HLA-DR

**Figure 6:**
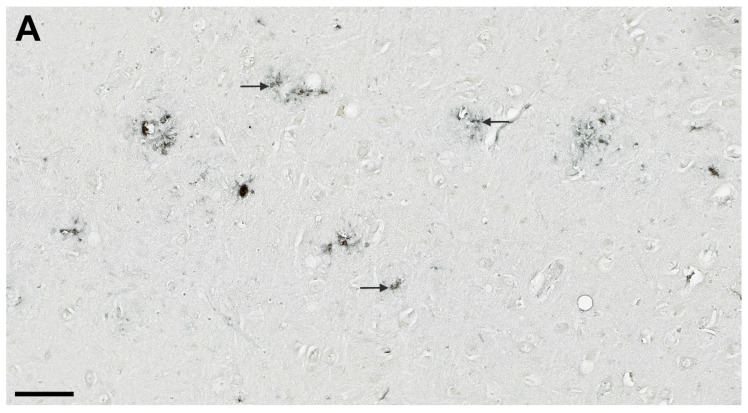

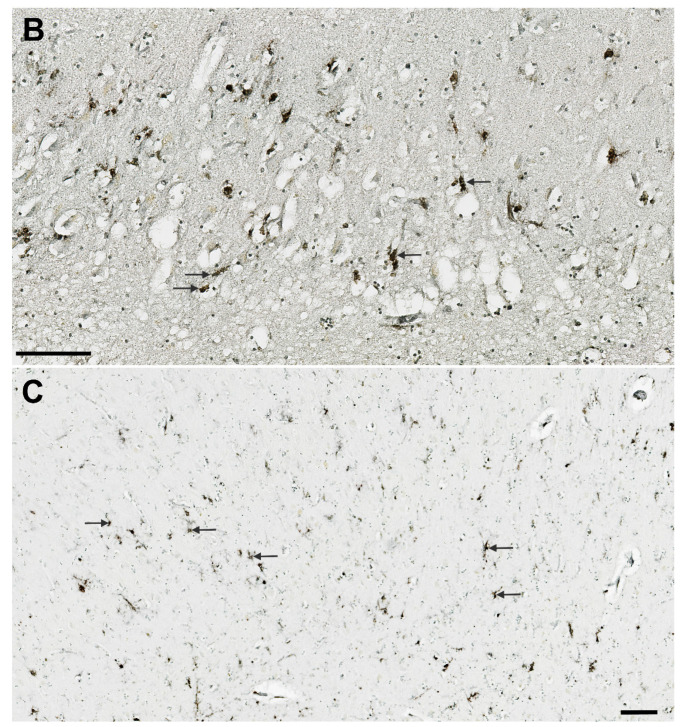
HLA-DR immunohistochemical staining of microglia (arrows) in AD. Hilar part of the CA3 (**A**), CA2/3 (**B**), and subiculum (**C**). The higher magnifications in A and B allow for the recognition of different morphologies of microglial cells, while panel C offers a broader view of their regional distribution. Scale bars 100 µm.

HLA-DR characteristically showed strongly activated microglial cells whose somas were irregularly thickened and of high signal intensity (Figure 6), in contrast to IBA1, which typically labels microglia with long and highly branched processes (Figure 5). Despite the trend of higher expression of HLA-DR in the AD group, the measured difference was not statistically significant (Table 5, Figure 4). The only significant quantitative difference between AD and HC was found in the CA1, where the overall number of labeled microglial cells was greater in the AD group (*p* = 0.003, Table 5). In both HC and AD groups analyzed together, HLA-DR immunoreactivity was correlated with the CD68 labeling in the CA1 and subiculum. While HC had a higher number of HLA-DR-immunopositive microglial cells in the subiculum and CA2/3, the AD cases had more HLA-DR-expressing microglial cells in the CA1 followed by the subiculum, and the lowest number in CA2/3 (Table 5). The most significant correlation of HLA-DR expression was with CASP-6 expression in the CA1 field (r_S_ = 0.62, *p* = 0.004, Table 2).

#### 3.6.3. CD68

**Figure 7:**
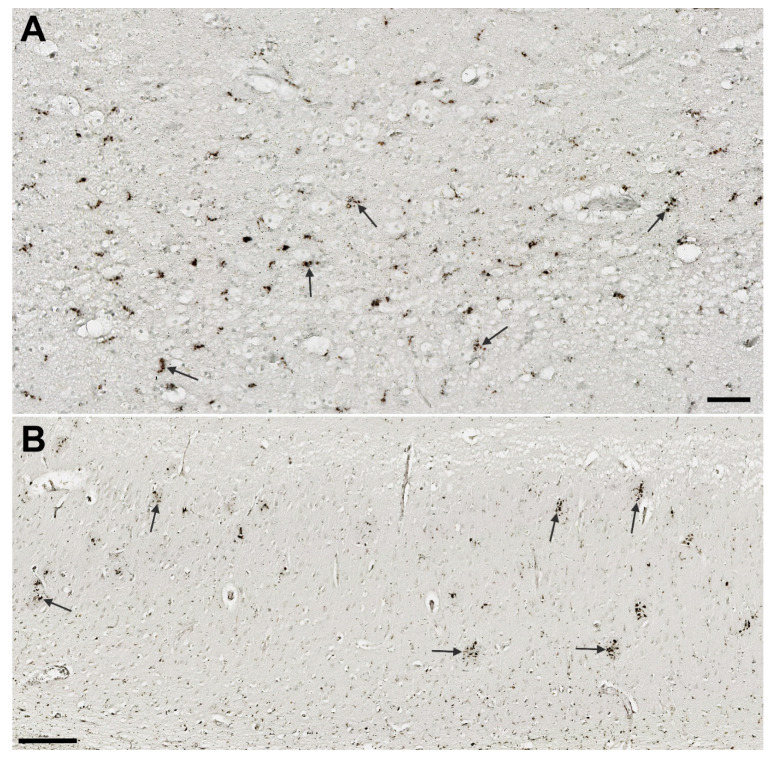
CD68 immunoreactivity of microglia. (**A**) Typical punctiform staining of CD68-labeled activated microglial cells (arrows) in a healthy control in the subiculum. (**B**) Clusters of CD68-expressing microglial cells (arrows) in the CA1 field in AD. Scale bars: A = 50 µm, B = 250 µm.

For CD68, we used the same 0-4 semiquantitative scale, but as CD68 is not a suitable marker for the morphological characterization of microglia, this time with slightly different descriptions. Essentially, in our samples, CD68 was seen predominantly as a diffusely distributed pointing signal or in a form of clusters, and we assessed the apparent local density of those typical clusters of punctate immunoreactivity patterns that reflect strong microglial activation (Figure 7). CD68 expression was significantly higher in the AD group throughout the HF (Table 5, Figure 4), and especially in CA1 and subiculum (*p* < 0.0001, Table 5). The highest signal in the AD group was in the CA1 while the lowest was in the CA2/3. Contrary, in the HC group, CA2/3 and hCA3 had the highest CD68 signal, whereas CA1 was the region with the lowest signal intensity. CD68 labeling was correlated with IBA1 in the CA2/3, and with HLA-DR in the CA1 and subiculum. CD68 expression was also positively correlated with NLRP1 (r_S_ = 0.45, *p* = 0.04) and CASP-6 (r_S_ = 0.76, *p* = 0.0001) in the CA1, and with NLRP1 (r_S_ = 0.61, *p* = 0.004), CASP-6 (r_S_ = 0.65, *p* = 0.002), and ASC (r_S_ = 0.5, *p* = 0.03) in the subiculum (Table 2).

None of the microglial markers (Iba 1, HLA-DR, CD68) correlated with the AD duration.

### 3.7. Illustrations of the Most Significant Findings

**Figure 8:**
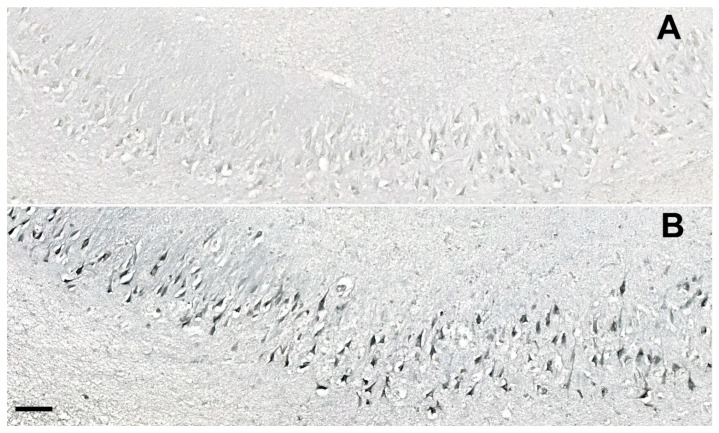
Immunostaining of NLRP1 in the CA2/3 field of the hippocampal formation in a control (**A**) and AD (**B**). See Figure 1 for quantitative data. Scale bar (same for A and B) = 100 µm.

**Figure 9:**
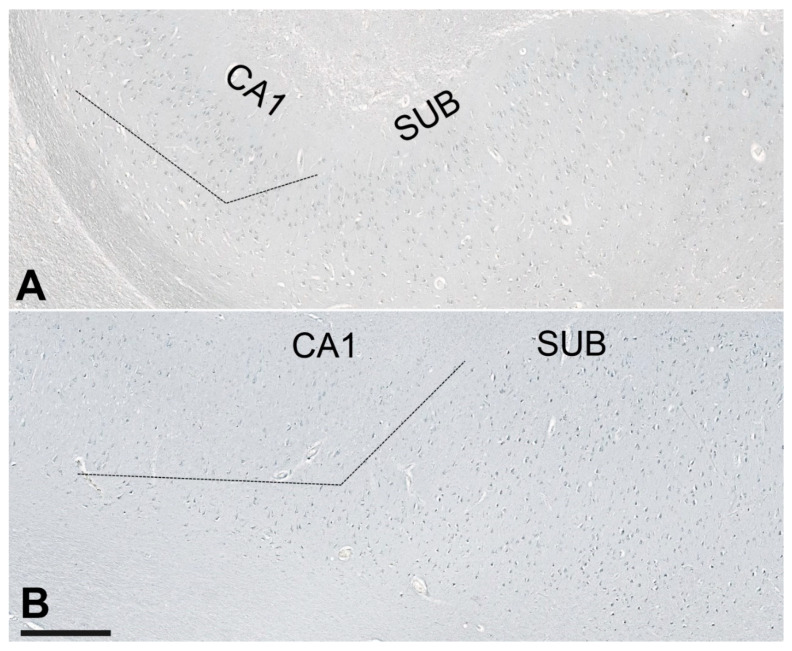
Immunostaining of ASC in the subiculum in control (**A**, case HC5) and AD brain (**B**, case AD2). The dashed line is a provisional border between the CA1 field and subiculum. The difference in the number of NLRP1-immunoreactive neurons between the HC and AD groups in the subiculum was statistically significant (see Figure 2). Scale bar (same for A and B) = 500 µm.

Compared to HC, the NLRP1 staining was always present in the many neurons in AD cases throughout the hippocampal formation, and especially in the CA2/3 field (Figure 8). Immunostaining of the NLRP1 in the CA2/3 field in HC (case 9) and AD (case 11) brain. The difference in the number of NLRP1-immunoreactive neurons between the HC and AD groups in the CA2/3 field was statistically significant (see Figure 1). The ASC immunohistochemical staining showed a significantly higher quantity of labeled cells in the subiculum in the AD group (Figure 9), but comparisons between the HC and AD groups in hCA3, CA2/3, and CA1 were not significant as was the hippocampal formation viewed as a whole.

**Figure 10:**
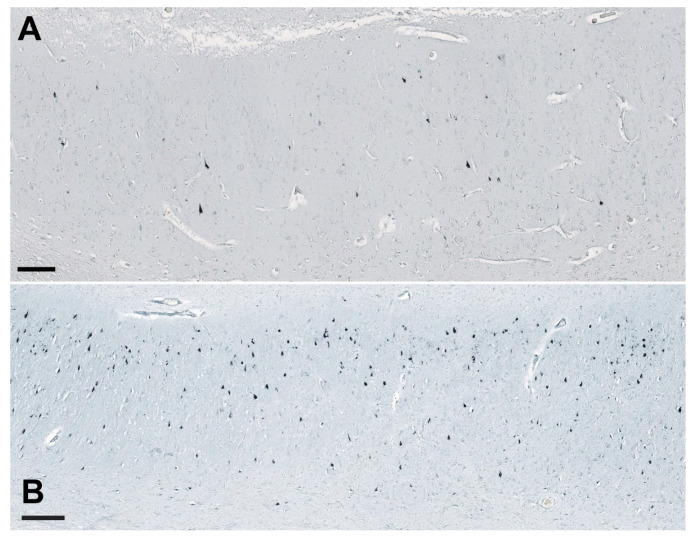
Immunostaining of the CASP-6 in the subiculum of the hippocampal formation in the HC (case HC9, subfigure **A**) and the AD brain (case AD2, subfigure **B**). The difference in the number of CASP-6-immunoreactive neurons between the HC and AD groups in the CA1 field was statistically significant (see Figure 4). Scale bars 250 µm.

The cGSDMD staining was strongest in the CA2/3 region and lowest in the subiculum, but differences among respective hippocampal fields were not significant. The strongest CASP-6 staining was found in the CA2/3 region in both HC and AD cases. A highly significant difference was found in the CA1 field, where AD cases had many more strongly CASP-6-immunoreactive neurons than HC (illustrated in Figure 10). Only rare and weakly CASP-6 immunoreactive neurons were present in the subiculum and CA1 field in the HC group.

### 3.8. Double-Labeling Experiments

Costainings were performed for the following combinations: NLRP1 and ASC, NLRP1 and AT8, NLRP1 and CD68, NLRP1 and HLA-DR, CASP-6 and AT8, CASP-6 and CD-68, CASP-6 and HLA-DR, ASC and AT8, and ASC and HLA-DR.

#### 3.8.1. Colocalization of NLRP1 with ASC, AT8, CD68, and HLA-DR

**Figure 11:**
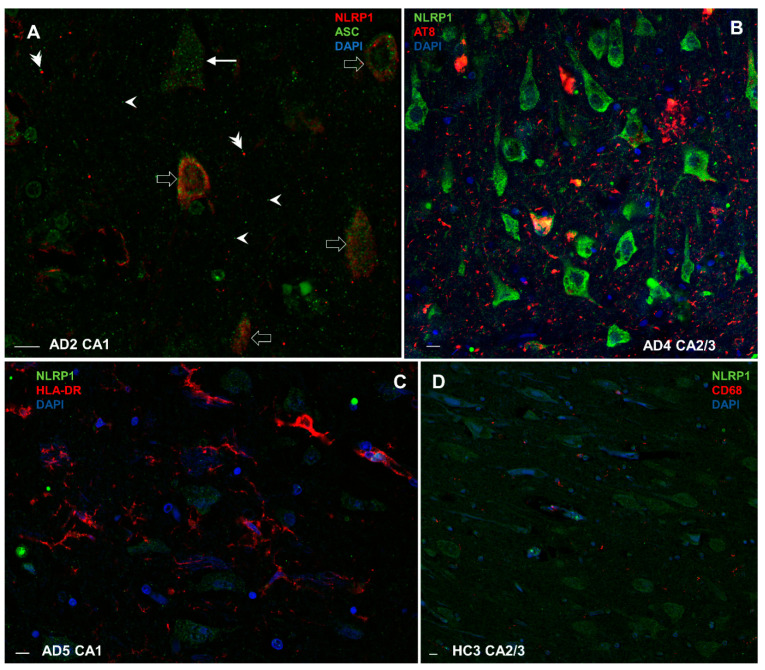
Colocalization of NLRP1 with ASC, phosphorylated tau (AT8), CD68, and HLA-DR in Alzheimer’s disease (**A**–**C**) and healthy control hippocampus (**D**). Open arrows in **A** show neurons in which NLRP1 inflammasome colocalizes with ASC protein. Full arrow in A shows presence of ASC proteins in a neuron in absence of NLRP1 inflammasome. Arrowheads in A show larger and brighter ASC signals that probably correspond to aggregates of self-oligomerized ASC protein, which are sometimes in the extracellular spaces of AD brains in association with NLRP1 (double arrowheads in **A**). AD brains generally show much higher expression of NLRP1 inflammasome (**A**,**B**) and HLA-DR-immunoreactive microglial cells (**C**) than healthy control brains (**D**). Scale bars = 10 μm.

NLRP1 inflammasome in some neurons colocalized with ASC protein (open arrows in Figure 11A), but in some ASC, protein was present in absence of NLRP1 inflammasome (e.g., full arrow in Figure 11A). Larger and brighter ASC signals probably correspond to self-oligomerized ASC protein-forming aggregates. Such aggregates were occasionally also found in the extracellular space of AD brains (arrowheads in Figure 11A), sometimes in association with NLRP1 (double arrowheads in Figure 11A). AD brains generally showed much higher expression of NLRP1 inflammasome (Figure 11A,B) and HLA-DR-immunoreactive microglial cells (**C**) than healthy control brains (Figure 11D).

#### 3.8.2. Colocalization of ASC with AT8

**Figure 12:**
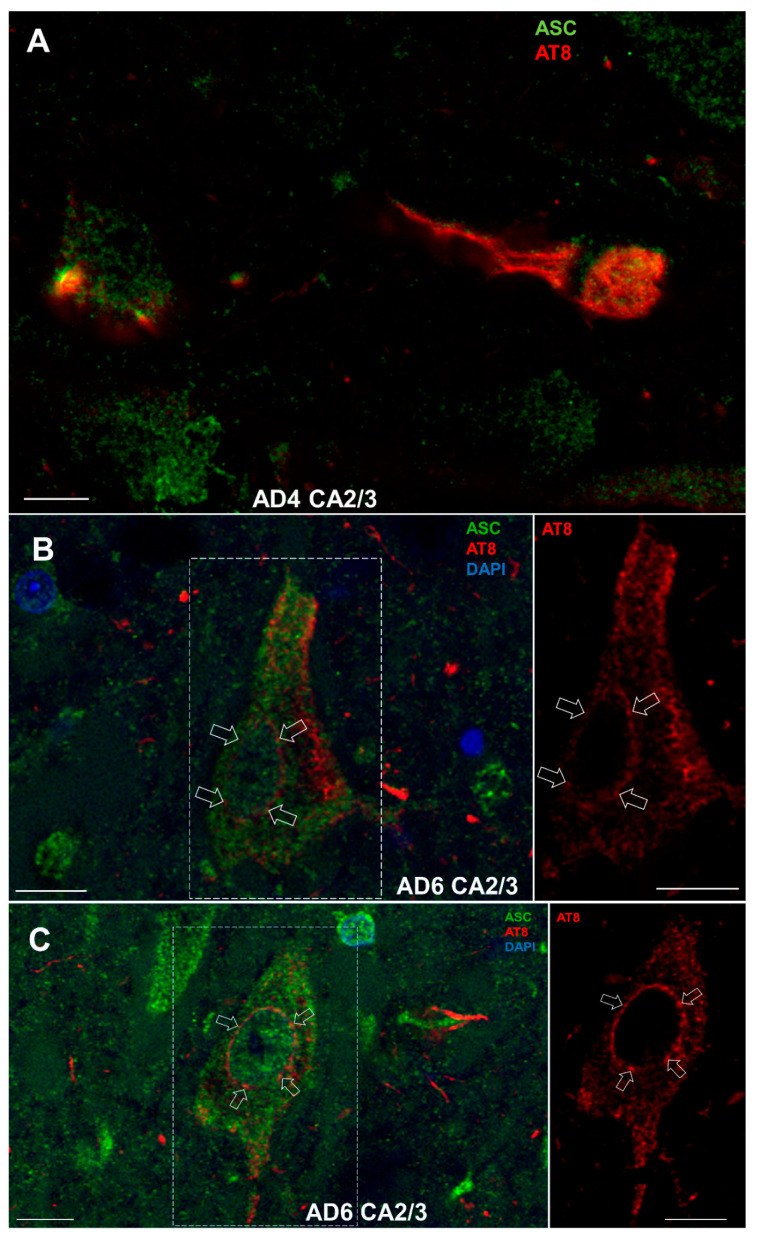
(**A**)**.** ASC protein colocalization with tau protein phosphorylated at Ser202 and Thr205 residues (AT8 epitope). Accumulation of AT8-immunoreactive tau proteins was observed at nuclear pores of large pyramidal neurons in the Ammon’s horn (**B**,**C**). Scale bars = 10 μm.

ASC protein very often colocalized in neurons with tau protein phosphorylated at Ser202 and Thr205 residues (AT8 epitope) (Figure 12A–C). The most striking observation was the accumulation of AT8-immunoreactive tau proteins at nuclear pores of large pyramidal neurons of the Ammon’s horn (Figure 12B,C).

#### 3.8.3. Colocalization of CASP-6 with AT8, CD68, and HLA-DR

**Figure 13:**
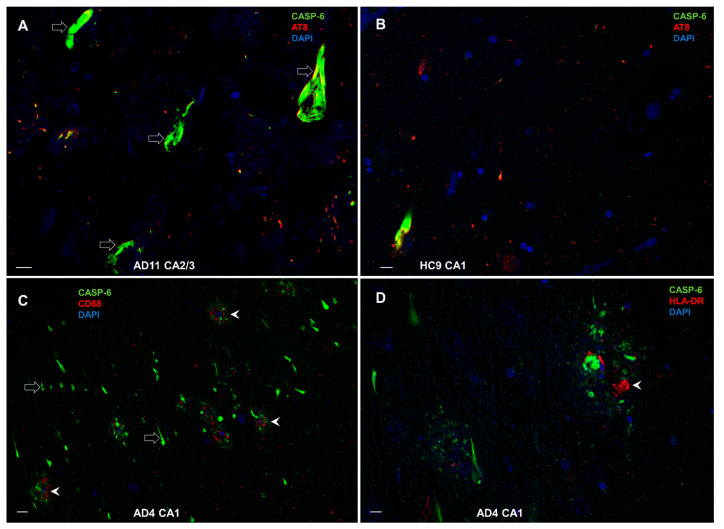
Colocalization of CASP-6 with phosphorylated tau (AT8), CD68, and HLA-DR. (**A**) CASP-6 and phosphorylated tau (AT8) in NFTs (empty arrows) in AD. (**B**) Colocalization is rare in HC, where NFTs are few. (**C**) CASP-6 and CD68 near amyloid/neuritic plaques in AD hippocampus (arrowheads). (**D**) The distribution and colocalization of CASP-6 and HLA-DR in AD are also prevalent in amyloid/neuritic plaques (arrowhead). Scale bars in A, B, and D = 10 μm, in C = 50 μm.

We found that CASP-6 and phosphorylated tau (AT8) highly colocalize in NFT (empty arrows in Figure 13A) in the AD brain, whereas the same can be seen in the brains of HC (Figure 13B), but on rare occasions, because there the amount of NFT is significantly lower. The distribution and colocalization of CASP-6 with CD68 and HLA-DR in the AD hippocampus were mainly found close to amyloid/neuritic plaques (arrowheads in Figure 13C and Figure 13D, respectively).

## 4. Discussion

The best-studied and characterized inflammasome is the NLRP3 inflammasome expressed mainly in microglial cells. In contrast, the NLRP1 inflammasome is predominantly expressed in pyramidal neurons and oligodendrocytes [38]. As both neurons and microglial cells are long-lived, they are sensitive to oxidative stress and inflammatory insults. Over decades of immune surveillance and stress responses, microglial cells may become hyper-reactive. Consequently, baseline expressions of inflammatory cytokines IL-1β, TNF-α, and IL-6 increase in microglia and brain tissue with age [49]. This suggests that inflammasomes, as immune sensors of a diverse array of signals, are responsible for a global increase in neuroinflammation with age. When the Nlrp3 inflammasome is deleted, NF-kB, IL-1β, interferon, and complement pathways are significantly attenuated in old Nlrp3 knockout mice compared to wild-type aged mice, suggesting that the Nlrp3 inflammasome is an upstream target that controls age-associated neuroinflammation [50]. Microglia are sensitive to weak repeated stimuli. Their priming has been described for various inflammatory markers, including scavenger receptor CD68. Consequently, microglia may express many macrophage-associated markers, such as CD11b, CD14, CX3C chemokine receptor 1, IBA1, and others, and generate a high amount of NLRP3 inflammasomes and pro-inflammatory cytokines and chemokines, such as IL-1β, IL-6, IL-12, TNF-α, CCL2, CXCL10, IL-18, nitric oxide, and others [3,51]. Damage- or pathogen-associated molecules stimulate resting microglial cells via membrane-bound or vesicular (endosomal) pattern-recognition receptors such as toll-like receptors (TLR), and NOD-like receptors (NLR), triggering receptors expressed on myeloid cells (TREM), RIG-I-like receptors (RLR), and others. Numerous studies have established that Aβ and tau proteins may trigger microglial activation by changing the microglial epigenome, transcriptome, proteome, metabolome, and phenome resulting in a specific morphological and functional outcome [51]. Therefore, potential overactivation of the inflammasomes may represent an initial event in AD early pathogenesis. A special subtype of microglia, dark microglia, characterized by condensed cytoplasm and nucleoplasm and pronounced chromatin remodeling to increased oxidative stress, is rarely present in physiological conditions but is seen in high numbers during aging, chronic stress, lack of the *CX3CR1* gene expression, and in transgenic APP/PS1 mice [52]. It is more active than normal microglia and expresses IBA1, CD11b as well as the TREM2 receptor in the presence of Aβ. To maintain control of microglial activation, neurons produce several immunomodulatory molecules that interact with microglia. These include membrane glycoprotein CD200, fractalkine ligand CX3CL1, as well as various neurotrophins and other molecules. The investigation of the NLRP1 inflammasome gains even more importance when knowing that *NLRP1* gene variants are associated with AD [28], whereas its silencing improves cognitive abilities and has a protective effect on neurons in animal models of AD [30,39,40,41,42]. In this study, we focused on the NLRP1 inflammasome, which had not been thoroughly analyzed before in AD, and to further assess the possible role of the inflammasome in AD. 

### 4.1. NLRP1 and ASC Immunoreactivity

Our results demonstrate that NLRP1 and ASC protein immunoreactivity are significantly higher in many more neurons in the AD hippocampus compared to controls, for NLRP1 especially in the CA2/3 field and for ASC especially in the subiculum. The reason for the strong NLRP1 activation in the AD brain is not known. It is possible that natural pathogen-derived effectors such as viral proteases, which can cleave human NLRP1 within a rapidly evolving region of the protein, lead to host-specific and virus-specific activation of the NLRP1 inflammasome [53]. Even in the case of eventual viral demise, the activated inflammasomes may be involved in later neurodegenerative changes. Other possibilities include direct or indirect activation of NLRP1 inflammasome due to cell stress caused by the AD pathological changes [23,30], predisposition given by certain gene polymorphisms [28], and prior activation of NLRP3 inflammasome in microglia [33]. One of the latest reports demonstrated that trazodone, an antidepressant with hypnotic efficacy in dementia, can reduce disease-related cellular pathways, including the NLRP3 inflammasome expression, and improve memory and sleep in male rTg4510 mice with a tauopathy-like phenotype [54].

Compared to ASC, our results show that NLRP1 immunostaining in the AD group is much stronger, possibly as the result of different NLRP1 activation pathways being involved. NLRP1 could activate CASP-1 with or without the recruitment of ASC [55]. Through its caspase activation and recruitment domain (CARD), NLRP1 can either bind the CASP-1 molecule directly, or in presence of ASC, exacerbate and amplify the activation of the CASP-1 [55]. As such, not every activated NLRP1 inflammasome involves ASC, explaining the observed differences in NLRP1 and ASC immunostaining. NLRP1 and ASC’s presence is correlated with their simultaneous involvement in the NLRP1 activation process. These observations are supported by studies in animal and in vitro models showing elevated NLRP1 expression in the brain or within cells with AD-like pathology [11,23,30]. Comparable findings were also reported in human AD, with the subiculum having many more NLRP1-immunoreactive neurons than controls, and *NLRP1* mRNA levels in the cerebral cortex in AD being higher relative to normal brains [23]. Compared to HC, NLRP1 was also much more strongly expressed in the subiculum in AD cases of our series, but—probably due to the small sample size—did not reach statistical significance. Overall, our results indicate that the NLRP1 inflammasome is likely involved in characteristic AD pathological changes, but the precise role and effects of the inflammasome activation in the development and progression of AD are complex and further investigations are needed.

### 4.2. Cleaved Gasdermin and Caspase-6 Immunoreactivity

In our materials, immunostaining of the cGSDMD had the weakest signal of all markers and is comparable in AD and control brains. In addition, cGSDMD expression predominates in the CA2 field. Although the CA2 field is considered resistant to neurofibrillary degeneration in aging and AD [56,57], some cases have selective neurofibrillary degeneration in CA2 with sparing of the more vulnerable CA1 field. These atypical cases are most commonly related to 4R tauopathies, such as argyrophilic grain disease [58,59] and other less common AD tauopathies [60] or cases with concomitant Lewy pathology [61]. Therefore, cleavage of the GSDMD in the hippocampal-formation neurons might be more related to general brain aging and genetic predisposition to distinct tauopathies than to AD *per se*. Alternatively, CASP-1 could have a higher affinity for CASP-6 in AD brains resulting in less cleavage of GSDMD. Our results contrast with previous findings of elevated GSDMD expression in AD or AD models [30,62,63,64], but these studies did not report GSDMD cleavage in the brain tissue. Moreover, most studies of neuronal pyroptosis have been performed in vitro or in animal models. Therefore, the cGSDMD signal in neurons indicates some physiological or pathophysiological change [65] but cannot be considered sufficient to confirm neuronal pyroptosis. It could, however, reflect upregulated cytokine release, because besides pyroptosis, GSDMD pores are also involved in the release of IL-1β and IL-18 [66] as well as IL-1α [67] after inflammasome activation. Levels of pro-inflammatory cytokines above the homeostatic range may contribute to the development or worsening of AD pathological changes [68]. Macrophages in culture have membrane-repairing survival mechanisms during pyroptosis, indicating that cleavage of the GSDMD does not necessarily imply that pyroptosis will occur [69], especially considering that neurons, as long-lived and terminally differentiated cells, are going through the slower process of degenerative changes [24,65,70,71,72]. The weak and regionally constrained cGSDMD signal in the AD brain, suggests that neurons in the human brain do not undergo pyroptosis as do cells of the immune system. cGSDMD presence in our study correlates with ASC and CASP-6 immunopositivity, implying the involvement in the inflammasome activation processes in the neurons. The role of GSDMD and the relationship between cytokine releasing and pyroptosis in neurons requires further investigation in human brain tissue.

Active CASP-6 immunostaining is the strongest of all markers analyzed in this study, and it is higher in the AD group compared to HC, in line with previous studies of AD brain tissues [23,25,26,71,73,74,75]. We show that neuronal CASP-6 immunoreactivity in the hippocampal formation positively correlates with the immunostaining for NLRP1 and ASC, which is congruent with its cleavage downstream of NLRP1 inflammasome activation in neurons [23]. Similar to GSDMD, CASP-6 expression in the brain in normal conditions is low. Therefore, any upregulation implies changes in the homeostatic state [76]. We document an active CASP-6 presence in the soma of hippocampal neurons. In contrast to NLRP1, ASC, and cGSDMD, whose expression is highest in the CA2/3 region, CASP-6 immunostaining is, besides CA2/3, also high in the CA1 in the AD group. The CA1 field is known to have a higher tau pathology burden [77,78,79] so we propose that because NLRP1, ASC, and cGSDMD have lower expression in the CA1 than CASP-6, they could be more active at the beginning of the pathological process, inside neurons at the early stage of neurofibrillary changes. Further, CASP-6 would also be present in later stages of AD in neurons with mature tangles. The strong CASP-6 staining in the CA1 of the AD group is compatible with the finding that higher levels of CASP-6 activity in the CA1 in aging correlate with lower cognitive performance [80].

### 4.3. Relationship of Inflammasome Activation with Neuron Loss, Neurofibrillary Pathology, and Other Indices of Neurodegeneration

The CA2/3 region has the highest immunoreactivity for all markers analyzed in both AD and HC groups. Whether inflammasome activation in AD is a cause or the consequence of the pathological processes remains unclear. In an earlier study, CA2/3 region neurons showed high neuronal nitric oxide synthase (nNOS) immunoreactivity and most of the neuron somata were spared from the neurofibrillary pathology [57]. Together with the data presented here, this implies that the higher production of NO and NLRP inflammasome activation are likely associated. In this context, constitutive nitric oxide synthases (nNOS, encoded by the *NOS1* gene on chromosome 12; inducible iNOS, microglial NOS, encoded by the *NOS2* gene on chromosome 17; and endothelial eNOS, encoded by the *NOS3* gene on chromosome 7) would contribute to the NO-mediated activation of inflammasomes. Our results also indicate that while NLRP1 inflammasome can exacerbate or cause AD-related pathological changes, the CA2/3 neurons may be more resistant to NLRP1-mediated tau pathological changes. The reason for the CA2/3 neurons resistance could be related to different microglial-neuronal and vascular interactions specifically in that region [81,82,83]. Some studies reported that NO can suppress NLRP3 inflammasome activation [84,85] and it is known that higher NLRP3 inflammasome activation can impact tau protein hyperphosphorylation, aggregation, and spreading [33,86,87]. NLRP3 in the brain is predominantly expressed in microglia and if it were suppressed in the CA2/3, those microglial cells could be switched from proinflammatory to their protective/scavenging state. Interestingly, a study on the ischemic brain has shown that the numbers of active astrocytes and microglia around damaged neurons is higher in the CA2/3 than the CA1 and proposed that this enhanced efficacy for eliminating damaged neurons has a neuroprotective effect [88]. Further, CA2/3 neurons are more receptive to the protective effects of the glial cell line-derived neurotrophic factor than CA1 neurons [81]. It will be important to further investigate the interactions between microglial (NLRP3) and neuronal (NLRP1) inflammasomes.

In the present study, no inflammasome marker correlated with the age of the subjects analyzed. Even though the age range was 59 to 91 years, age-related differences in NLRP1 inflammasome activation were not observed. Although the sample size in our study is relatively small, this contrasts with a recent study in which NLRP1 activation in mouse brains was closely associated with aging-related brain changes [89]. Similarly, it has been reported that NLRP1 activation is stronger in female APP/PS1^+/−^ mice brains with AD-like pathology [11], whereas we observed no significant correlation between any investigated marker with sex. Hence, age and sex differences in NLRP1 inflammasome activation may differ considerably among species. It can also be concluded that, as long as elderly individuals do not suffer from AD, they appear neuropathologically quite comparable as a group [77] and significant changes related to aging cannot be revealed without the inclusion of younger cases in the regressions [90].

Our results show that the total density of NLRP1-immunoreactive neurons in the HF significantly positively correlates with the total number of NFTs, suggesting that NLRP1 inflammasome activation is likely associated with tau-related pathology. This hypothesis has been already proposed based on NLRP1 and tau cleaved by CASP-6 (TauΔCasp6) co-expression in the same neurons [23]. Still, the role of the inflammasome in the development or progression of AD has yet to be fully elucidated.

### 4.4. Relationship of Inflammasome Activation with Microglial Markers IBA1, HLA-DR, and CD68

A partially overlapping expression of microglial markers has been reported in both normal brain tissue and disease conditions [91,92,93,94]. Our results show positive correlations of NLRP1 inflammasome with microglial IBA1 expression in the subiculum and with microglial CD68 expression in the CA1 and subiculum in AD. The HLA-DR microglial expression is highly correlated with CASP-6 expression in the CA1, whereas CD68 microglial expression is correlated with CASP-6 expression in the CA1 and subiculum. As revealed by CD68 and HLA-DR markers, the positive correlations of microglial activation with NLRP1 and CASP-6 immunoreactivity indicate that stronger microglial activation in these regions may induce stronger NLRP1 inflammasome activation and accelerated neurofibrillary degeneration. We also show that AT8-immunoreactive phosphorylated tau proteins accumulate at nuclear pores in large pyramidal neurons of the hippocampal Ammon’s horn, thus directly impairing nuclear transport. This finding supports the hypothesis that pathological tau proteins can directly interact with nuclear pore complex components, leading to their mislocalization and consequent disruption of nuclear pore complex function [95].

The finding that other patterns of inflammasome and microglial activation expression did not reach statistical significance may be related to the fact that in AD cases many neurons and synapses are already lost, which affects expression levels and immunoreactivity. Hence, our findings strongly support neuroinflammation as one of the primary drivers of AD-related neuropathological changes, neuron and synapse loss, as well as corresponding cognitive and behavioral deficits. This interpretation is also in good agreement with the latest two-stage genome-wide association study on 111,326 clinically diagnosed AD cases and 677,663 HC, where pathway enrichment analyses confirmed and expanded on a causal involvement of amyloid precursor protein (APP), tau, and tau-binding proteins in AD pathogenesis, at the same time highlighting the key role of microglial activation and the likely involvement of microglial endocytosis, a mechanism that is also heavily involved in APP metabolism [96]. As the early, repeated, and extensively prolonged microglial proliferation observed in AD probably also endangers their transcriptional and phenotypic trajectory, it also promotes replicative senescence of these cells, characterized by increased β-galactosidase activity, telomere shortening, a senescence-associated transcriptional signature that correlate with the appearance of disease-associated microglia and senescent microglial profiles in human postmortem AD cases [97].

### 4.5. Limitation of the Study

The blood and cerebrospinal fluid biomarkers of HC and AD were not available for present cohort, preventing us from providing such correlates of pathology.

## 5. Conclusions

Our results show that NLRP1, ASC, and CASP-6 markers of NLRP1 inflammasome activation are more strongly expressed in the HF in AD brains compared to controls. The expression of different markers of NLRP1 inflammasome activation and microglial activation markers was unrelated to age and disease duration. Interestingly, all markers have the highest immunoreactivity in the CA2/3 region, whose pyramidal neurons exhibit resistance to NFT formation, suggesting that the CA2/3 neurons are more resistant to both neurofibrillary pathology and inflammasome activation-related changes than other pyramidal cells of the hippocampus and neocortex. While our results confirm some previous reports of increased NLRP1 expression in AD, they also reveal new information. First, NLRP1 is expressed in significantly more CA2/3 neurons in the AD brain compared to controls, while ASC is expressed in significantly more subicular neurons in the AD brain. CASP-6 is expressed in significantly more CA1 neurons in the AD brain than in controls. Interestingly, markers of NLRP1 inflammasome activation in the HF do not correlate with the age and the duration of AD but the total number of NLRP1-immunoreactive neurons per area of hippocampal formation tissue is positively correlated with the total number of NFTs. Moreover, and somewhat unexpectedly, the number of NFTs did not correlate with the age of the AD subject in any of the HF fields analyzed, except in the CA1, where it was negatively correlated with age, meaning that younger AD patients had more NFTs in the CA1 field than older ones. Finally, in contrast to our findings, a report on activated microglia density in the chimpanzee brain showed that microglial activation was not significantly correlated with neurofibrillary lesions composed of hyperphosphorylated tau proteins [98]. This indicates that the chimpanzee brain may be relatively well protected during normal aging and that susceptibility to inflammasome activation may render the human brain comparatively more vulnerable to neurodegenerative changes and AD. In conclusion, although the precise roles of NLRP1 inflammasome activation in the development and progression of AD and other tauopathies have yet to be fully elucidated, our findings reveal region-specific mechanisms of NLRP1 inflammasome activation in the HF and suggest that its suppression represents a valid therapeutic goal for the treatment or prevention of AD.

## Figures and Tables

**Table 1:** Demographic data of control and AD subjects.

**Case**	**HC**				
	**Age**	**Gender**	**Cause of Death**		
**HC1**	59	M	Car accident		
**HC2**	62	F	Car accident		
**HC3**	68	M	Car accident		
**HC4**	71	F	Myocardial infarction		
**HC5**	75	M	Car accident		
**HC6**	77	F	Myocardial infarction		
**HC7**	81	F	Myocardial infarction		
**HC8**	84	F	Pulmonary embolism		
**HC9**	85	F	Cardiovascular failure		
**Case**	**AD**				
	**Age**	**Gender**	**Duration of the Disease (years)**	**Cause of Death**	**NINCDS-ADRDA Diagnosis**
**AD1**	73	F	4	Bronchopneumonia	Definitive AD
**AD2**	73	M	7	Bronchopneumonia	Definitive AD
**AD3**	77	M	3.5	Bronchopneumonia	Definitive AD
**AD4**	80	F	5	Bronchopneumonia	Definitive AD
**AD5**	80	F	6	Myocardial infarction	Definitive AD
**AD6**	81	M	3	Bronchopneumonia	Definitive AD
**AD7**	83	F	5.5	Cardiovascular failure	Probable AD
**AD8**	84	F	3.5	Cardiovascular failure	Definitive AD
**AD9**	84	F	3.5	Cardiovascular failure	Definitive AD
**AD10**	88	F	4.5	Carcinoma	Probable AD
**AD11**	91	F	4	Bronchopneumonia	Probable AD

**Table 2:** Results of NLRP1 (N), ASC (A), cGSDMD (G), and CASP-6 (C) immunostaining assessment. Numbers represent the total number of all immunoreactive neurons in a given hippocampal subdivision in a randomly selected section of the hippocampus.

Case	hCA3 Counts				Area	CA2/3 Counts				Area	CA1 Counts				Area	SUB Counts				Area	Total Counts				Σ Area
	N	A	G	C	mm^2^	N	A	G	C	mm^2^	N	A	G	C	mm^2^	N	A	G	C	mm^2^	N	A	G	C	mm^2^
**HC1**	4	12	0	12	5981	23	26	0	1	3449	113	140	0	84	8421	0	0	0	0	0	140	178	0	97	17,852
**HC2**	86	92	32	108	7837	0	0	1	23	0	16	17	3	51	10,702	63	53	4	55	14,885	165	162	40	237	33,424
**HC3**	16	19	32	9	7738	38	24	47	11	11,277	30	50	50	9	17,299	67	32	22	55	12,825	151	125	151	84	49,139
**HC4**	52	38	10	13	3953	188	139	24	75	2302	159	100	4	35	10,149	279	121	2	44	10,065	678	398	40	167	26,468
**HC5**	54	95	81	98	7362	183	200	185	340	6829	113	109	252	230	22,223	57	155	43	55	23,678	407	559	561	723	60,092
**HC6**	35	17	12	7	4201	45	20	9	9	1803	28	18	3	14	13,001	65	173	48	70	10,486	173	228	72	100	29,490
**HC7**	6	149	17	43	4486	74	159	30	70	1596	44	131	11	22	12,506	96	160	41	53	9121	220	599	99	188	28,108
**HC8**	16	41	4	13	3445	32	149	8	78	1388	19	119	14	20	6647	20	96	0	15	15,423	87	405	26	126	26,903
**HC9**	73	83	7	13	5412	67	36	0	5	3916	69	26	0	16	14,360	34	42	0	24	14,239	243	187	7	58	37,926
**AD1**	58	109	15	114	6247	73	129	51	102	2162	28	39	14	124	6441	24	99	13	146	6131	183	376	93	486	20,980
**AD2**	60	278	48	90	3897	127	124	58	80	1180	104	36	10	141	4680	284	300	36	129	9659	575	738	152	440	19,416
**AD3**	7	123	26	33	4018	96	81	45	41	2619	16	32	6	70	11,055	44	60	13	10	11,419	163	296	90	154	29,111
**AD4**	88	74	23	44	12,189	200	113	8	50	2671	180	27	2	171	7269	179	119	0	170	15,626	647	333	33	435	37,754
**AD5**	38	54	10	23	7351	152	131	36	25	1571	79	100	38	207	7029	98	170	11	130	10,083	367	455	95	385	26,034
**AD6**	84	156	21	39	8899	148	121	7	39	2588	100	14	0	79	5571	132	142	9	40	7837	464	433	37	197	24,894
**AD7**	38	227	44	60	4881	141	278	60	69	2720	65	82	15	226	7956	257	146	8	97	7240	501	733	127	452	22,797
**AD8**	45	67	22	74	8738	151	78	17	29	3386	105	38	29	439	8296	67	29	12	210	6921	368	212	80	752	27,342
**AD9**	4	45	11	29	3622	19	88	44	35	1995	48	19	16	275	6168	6	74	14	180	7741	77	226	85	519	19,527
**AD10**	20	17	12	22	4356	187	84	27	61	4201	66	54	24	215	21,832	109	45	4	245	6501	382	200	67	543	36,890
**AD11**	32	140	23	63	7476	113	172	8	67	2562	87	52	5	25	6547	32	117	0	2	3276	264	481	36	157	19,862

**Table 3:** Densities of immunoreactive neurons for the four different markers per mm^2^ of hippocampal formation tissue.

Case	NLRP1 (Cells/mm^2^)	ASC (Cells/mm^2^)	GSDMD (Cells/mm^2^)	CASP-6 (Cells/mm^2^)
**HC1**	0.784	1.000	0.000	0.543
**HC2**	0.494	0.480	0.120	0.709
**HC3**	0.307	0.250	0.307	0.171
**HC4**	2.562	1.500	0.151	0.631
**HC5**	0.677	0.930	0.934	1.203
**HC6**	0.587	0.770	0.244	0.339
**HC7**	0.783	2.130	0.352	0.669
**HC8**	0.323	1.510	0.097	0.468
**HC9**	0.641	0.490	0.019	0.153
**AD1**	0.872	1.792	0.443	2.316
**AD2**	2.961	3.801	0.783	2.266
**AD3**	0.560	1.017	0.309	0.529
**AD4**	1.714	0.882	0.087	1.152
**AD5**	1.410	1.748	0.365	1.479
**AD6**	1.864	1.740	0.149	0.791
**AD7**	2.198	3.215	0.557	1.983
**AD8**	1.346	0.775	0.293	2.750
**AD9**	0.394	1.157	0.435	2.658
**AD10**	1.036	0.542	0.182	1.472
**AD11**	1.329	2.422	0.181	0.790

**Table 4:** The total number of neurons (NN) and neurofibrillary tangles (NFTs) in hippocampal formation domains in HC and AD subjects.

Case	hCA3		CA2/3		CA1		SUB		Σ
	NN (10^6^)	NFTs (10^6^)	NN (10^6^)	NFTs (10^6^)	NN (10^6^)	NFTs (10^6^)	NN (10^6^)	NFTs (10^6^)	NFTs (10^6^)
**HC1**	0.84	0	1.2	0	7.18	0	2.61	0	**0**
**HC2**	1.29	0	1.41	0	8.41	0	4.34	0	**0**
**HC3**	1.57	0	1.94	0	2.62	0	2	0	**0**
**HC4**	1.27	0	2.9	0	5.35	0	1.47	0	**0**
**HC5**	1.4	0	2.48	0	8.28	0	4.85	0.05	**0.05**
**HC6**	0.83	0	1.68	0	2.43	0	1.79	0.03	**0.03**
**HC7**	0.87	0	1.61	0	3.42	0	2.29	0	**0**
**HC8**	1.35	0	1.45	0	7.89	0.01	4.89	0	**0**
**HC9**	1.29	0	2.16	0	2.26	0	1.59	0	**0**
**AD1**	1	0.08	0.98	0.22	4.93	3.45	0.93	0.71	**4.45**
**AD2**	1.63	0.08	1.45	0.51	5.48	4.02	3.47	0.54	**5.14**
**AD3**	0.57	0.13	1.64	0	5	0.81	1.42	0.25	**1.19**
**AD4**	0.93	0.16	1.82	0.11	10.9	1.95	2.78	0.43	**2.66**
**AD5**	0.3	0	0.92	0.18	4.38	3.15	2.4	0.52	**3.85**
**AD6**	1.8	0.3	0.78	0.2	4.59	2.88	3.16	1.19	**4.57**
**AD7**	0.37	0	1	0	3.09	1.99	1.25	0.13	**2.13**
**AD8**	1.67	0.07	1.39	0.11	7.65	2.42	3.81	0.55	**3.15**
**AD9**	0.17	0.08	1.26	0	2.06	1.42	1.01	0.3	**1.8**
**AD10**	0.23	0	0.84	0.19	3.27	1.88	1.79	0.68	**2.74**
**AD11**	0.53	0.08	0.96	0.37	1.14	0.55	0.84	0.27	**1.27**

Bold numbers represent totals from the individual subdivisions.

**Table 5:** Results of the semiquantitative scale assessment of microglial markers IBA1 (I), HLA-DR (H), and CD68 (C).

Case	hCA3 Counts			CA2/3 Counts			CA1 Counts			SUB Counts			Total Counts per Sample		
	I	H	C	I	H	C	I	H	C	I	H	C	I	H	C
**HC1**	3	3	3	2	3	2	0	2	2	1	2	2	**6**	**10**	**9**
**HC2**	3	2	2	3	2	3	2	3	1	3	2	1	**11**	**9**	**7**
**HC3**	2	0	1	2	0	2	2	0	1	1	0	1	**7**	**0**	**5**
**HC4**	2	0	1	2	0	1	2	0	2	3	0	2	**9**	**0**	**6**
**HC5**	2	2	2	1	2	2	0	2	1	0	2	1	**3**	**8**	**6**
**HC6**	2	2	2	1	3	2	2	2	2	2	2	2	**7**	**9**	**8**
**HC7**	0	1	2	1	0	2	1	0	1	0	2	3	**2**	**3**	**8**
**HC8**	3	0	2	2	0	2	2	0	1	2	1	2	**9**	**1**	**7**
**HC9**	2	0	3	1	0	2	2	0	2	2	0	2	**7**	**0**	**9**
**AD1**	0	0	3	0	2	2	0	1	4	0	1	3	**0**	**4**	**12**
**AD2**	2	1	4	3	1	3	3	2	4	3	1	4	**11**	**5**	**15**
**AD3**	0	1	3	2	0	3	1	2	4	0	2	3	**3**	**5**	**13**
**AD4**	2	1	3	3	0	n.a.	2	3	4	3	4	4	**10**	**8**	**11**
**AD5**	3	3	3	2	3	3	3	3	4	3	3	3	**11**	**12**	**13**
**AD6**	2	2	4	3	0	3	3	3	4	2	2	3	**10**	**7**	**14**
**AD7**	0	2	1	3	0	3	2	4	3	4	4	4	**9**	**10**	**11**
**AD8**	0	4	4	1	3	3	1	4	4	0	4	4	**2**	**15**	**15**
**AD9**	0	2	4	3	0	3	3	2	4	2	0	3	**8**	**4**	**14**
**AD10**	3	0	2	3	2	3	3	3	3	3	2	4	**12**	**7**	**12**
**AD11**	3	3	2	3	2	2	3	3	2	2	3	3	**11**	**11**	**9**

Bold numbers represent totals from the individual subdivisions.

## Data Availability

All the data reported are available on request from the corresponding author.

## Abbreviations

The following abbreviations are used in this manuscript:

4R tau	tau proteins with four microtubule-binding domain repeats
AD	Alzheimer’s disease
Aβ	amyloid β
APP	amyloid precursor protein
ASC	apoptosis-associated speck-like protein
CA	cornu Ammonis
CARD	caspase activation and recruitment domain
CASP-1	caspase-1
CASP-6	caspase-6
CD68	cluster of differentiation 68
CERAD	Consortium to Establish a Registry for Alzheimer’s Disease
cGSDMD	cleaved gasdermin
DSM-IV	Diagnostic and Statistical Manual of Mental Disorders, fourth edition
HC	healthy controls
HF	hippocampal formation
hCA3	hilar part of the CA3 field
HLA-DR	Human Leukocyte Antigen–DR isotype
IBA1	Ionized calcium-binding adapter molecule 1
NFTs	neurofibrillary tangles
NINCDS-ADRDA	National Institute of Neurological and Communicative Diseases and Stroke/Alzheimer’s Disease and Related Disorders Association
NLRs	NOD-like leucine-rich repeat receptors
NLRP1	nucleotide-binding domain and leucine-rich repeat-containing receptor family, pyrin domain-containing protein 1
NLRP3	nucleotide-binding domain and leucine-rich repeat-containing receptor family, pyrin domain-containing protein 3
NN	number of neurons
NOD	nucleotide-binding and oligomerization domain
PBS	phosphate buffered saline
r_P_	Pearson’s correlation coefficient
r_S_	Spearman’s correlation coefficient
SD	standard deviation
SUB	subiculum
